# *In Vitro* and *in Vivo* Anticancer Activity of Aconitine on Melanoma Cell Line B16

**DOI:** 10.3390/molecules18010757

**Published:** 2013-01-08

**Authors:** Juan Du, Xiaonian Lu, Ziwen Long, Zhen Zhang, Xiaohua Zhu, Yongsheng Yang, Jinhua Xu

**Affiliations:** 1 Dermatology Department, Huashan Hospital, Fudan University, Shanghai 200040, China; 2 Shanghai Cancer Center, Fudan University, Shanghai 200040, China

**Keywords:** melanoma, B16, aconitine, apoptosis, PI3K/AKT, MAPK/ERK1/2

## Abstract

The anti-tumor effect of aconitine in melanoma cell line B16 has been studied in this paper. We found that B16 cells showed significantly reduced growth rates and increased apoptotic effects in the presence of aconitine. Furthermore, aconitine inhibited the PI3K/AKT and MAPK/ERK1/2 signaling pathways, thus regulating the levels of protein and mRNA of PCNA and apoptotic related signaling molecules. Above all, we found that aconitine showed an anti-melanoma effect in suppressing tumor growth *in vivo*. In conclusion, we show that aconitine may be a useful anticancer drug in the future.

## 1. Introduction

Melanoma is the main cause of death in patients with skin cancer around the World [1]. Melanoma is less common than other skin cancers, however, it is much more dangerous if it is not detected early, and is responsible for the majority (75%) of skin cancer-related deaths [2]. The spread of metastatic melanoma (MM) to other organs is one of the most dangerous conditions that is almost uniformly fatal for the majority of patients with the currently available treatment modalities. Since melanoma is an immunogenic tumor, developing novel immune strategies will continue to play a critical role in designing effective treatment modalities for those at high risk of recurrence and those with distant metastasis [3,4]. The treatment includes surgical removal of the tumor. If melanoma is found early when it is still small and thin, and completely removed, the chances of cure are high. The likelihood of the melanoma coming back or spreading out depends on how deeply it goes into the layers of the skin. For melanomas that come back or spread out, treatments include chemo- and immunotherapy, or radiation therapy [5,6].

Malignant melanoma is a highly aggressive tumor of the pigment-producing cells in the skin with a rapidly increasing incidence and a poor prognosis for patients with advanced disease that is resistant to current therapeutic concepts. Immunosuppression within and around tumors has been implicated as a major factor in preventing better clinical outcomes using cytokines such as interleukin-2(IL-2) and IFN-α [7,8,9]. Recent clinical trials suggested that dacarbazine (DTIC) in sequence with low-dose recombinant human interleukin-2 might be an efficacious and relatively non-toxic chemo-immunotherapeutic treatment, which may synergistically eliminate tumours [10], but clinical drugs have not shown strong therapeutic efficacy with low side effects [11,12]. According to the American Cancer Society, there are no established guidelines to prevent malignant melanoma.

Mounting evidence directly indicates aberrant activation of PI3K/AKT and MAPK/ERK1/2 signaling in malignant progression of a variety of human cancers such as human breast carcinoma, lung cancer, lymphomas, leukemias, and malignant melanoma [13,14,15,16]. In melanoma, both the Ras-Raf-MEK-ERK (MAPK) and the PI3K-AKT (AKT) signaling pathways are constitutively activated through multiple mechanisms, and thus exert several key functions in melanoma development and progression [17]. Extracellular signal-regulated kinase 1/2 (ERK1/2) and phosphatidylinositol 3-kinase (PI3K)/protein kinase B (Akt) signaling pathways are two important kinase cascades that mediate the invasion and metastasis of melanoma [18,19].

Aconitine was previously used as an antipyretic and analgesic and still has some limited applications in herbal medicine although the narrow therapeutic index makes calculating an appropriate dosage difficult. Recent reports have shown that aconitine could inhibit the growth and invasion of tumors originating from human breast cancer cell line MDA-MB-231BO by blocking the PI3K/AKT signaling pathway [20]. Until now, aconitine is not available in clinic, partly due to arguments about its toxicity and activity [21,22].

In the present study, we used the molecular-biological technology for the high-throughput screening of natural product to select specific drugs for melanoma, and then obtained aconitine that inhibited the growth of melanoma cell line B16 *in vitro*, and attenuated the growth of solid tumor in nude mice. These data indicate that aconitine has potential effects as anticancer agents.

## 2. Results and Discussion

### 2.1. Results

#### 2.1.1. B16 Mouse Melanoma Cells Were Sensitive to Aconitine

**Figure 1:**
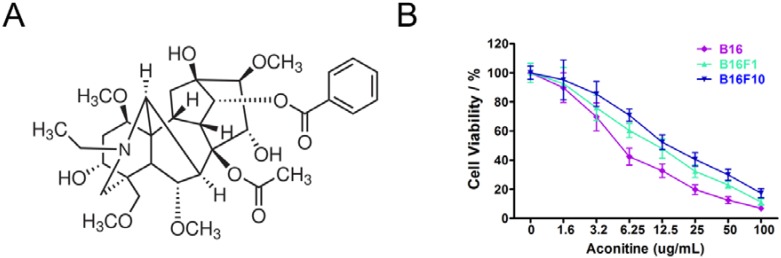
B16 melanoma cells were sensitive to aconitine. (**A**) Chemical structure of aconitine; (**B**) B16, B16F1 and B16F10 cells were treated with different concentration of aconitine ranging from 1.6 to 100 µg/mL for 48 h. Cell viability was determined by WST-1 method. Data were presented as means ± SEM from three separate experiments.

Aconitine is the most effective component of aconite (Figure 1A). A serial concentration of aconitine was prepared and used to treat melanoma cells B16 and its metastatic derivative cells B16F1 and B16F10. As shown in Figure 1B, B16 cells were more sensitive to the treatment of aconitine than its derivatives. IC_50_ values of B16 cells treated by aconitine for 48 h was 7.58 ± 0.99 µg/mL, while the IC_50_ values of B16F1 and B16F10 were 12.01 ± 1.12 µg/mL and 17.09 ± 1.03 µg/mL, respectively (Table 1). Taken together, B16 cells were more sensitive to aconitine than the other two cell lines, B16F1 and B16F10.

**Table 1:** IC_50_ values of aconitine treated melanoma cells for 48 h. * indicated significant differences in IC_50_ value between B16 and B16F1 cells, while ^#^ represented significant differences in IC_50_ value between B16 and B16F10. *, ^#^ *p* < 0.05.

Melanoma cell lines	IC50 (µg/mL)
B16	7.58 ± 0.99 *^,#^
B16F1	12.01 ± 1.12
B16F10	17.09 ± 1.03

#### 2.1.2. Aconitine Suppressed Cell Growth and Increased Apoptosis of B16 Cells

**Figure 2:**
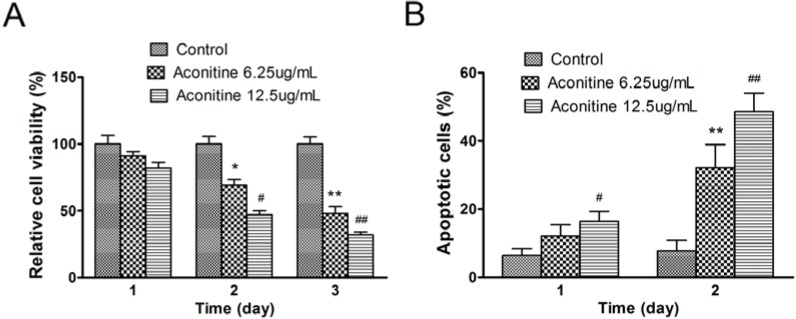
Aconitine inhibited cell growth and induced apoptosis of melanoma B16 cells. (**A**) Growth of B16 cells treated by aconitine at 6.25 µg/mL and 12.5 µg/mL, respectively; (**B**) Annexin-V analysis of apoptotic cells of B16 cells treated with aconitine at 6.25 µg/mL and 12.5 µg/mL, respectively. Data were presented as means ± SEM from three separate experiments. * *p* < 0.05, ** *p* < 0.001 represented that B16 cells treated with 6.25 µg/mL of aconitine compared to control (0 µg/mL of aconitine), while ^#^ *p* < 0.05, ^##^ *p* < 0.001 represented that B16 cells treated with 12.5 µg/mL of aconitine compared to control.

In order to confirm the inhibiting effects of aconitine on melanoma cell line B16 *in vitro*. Cells were seeded in 96-well plates and treated with or without aconitine. As shown in Figure 2A, aconitine inhibited growth of B16 cells. The high dose of aconitine (12.5 µg/mL) was much more effective than low dose of aconitine (6.25 µg/mL). The apoptosis of B16 cells induced by aconitine treatment was measured by FACS. In the presence of aconitine for 2 days, about 50% (high dose), 38% (low dose) of cells were apoptotic, which were significantly higher than those of control (Figure 2B).

#### 2.1.3. Aconitine Inactivated PI3K/AKT and MAPK/ERK1/2 Signaling Pathways

**Figure 3:**
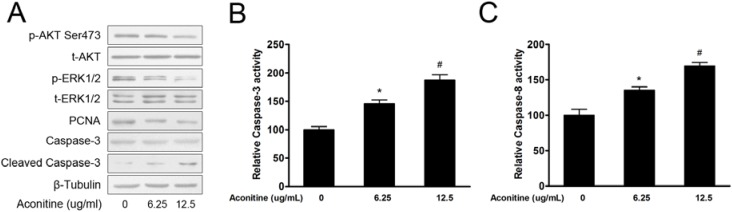
Aconitine inhibited cell proliferation and induced apoptosis by modulating AKT and ERK1/2 signaling pathway. (**A**) Western blotting analysis of effect of aconitine on activation of AKT and ERK1/2, protein expression of PCNA and Caspase-3. β-Tubulin was used as internal control. (**B**, **C**) Analysis of kinase activities of Caspase-3 (**B**) or Caspase-8 (**C**) when B16 were treated with aconitine at 6.25 µg/mL and 12.5 µg/mL, respectively. Those B16 cells not treated with aconitine were used as control. Data were means ± SEM from three separate experiments. * *p* < 0.05 represented that B16 cells treated with 6.25 µg/mL of aconitine compared to control (0 µg/mL of aconitine), while ^#^ *p* < 0.05 represented that B16 cells treated with 12.5 µg/mL of aconitine compared to control.

Activation of both PI3K/AKT and MAPK/ERK1/2 signalling pathway have been reported in human melanoma [17], which presented molecular targets for the effective treatment of advanced melanoma [23]. B16 cells were exposed to aconitine (6.25 µg/mL and 12.5 µg/mL) for 48 h. A Western blotting assay showed that aconitine inactivated AKT and ERK1/2, suppressed protein levels of PCNA, and induced cleave of Caspase-3 (Figure 3A). In addition, kinase activity analysis indicated that aconitine significantly activated both Caspase-3 and Caspase-8 (Figure 3B). Therefore, the result suggested that aconitine possibly inhibited these two signaling pathways to reduce B16 cell growth.

#### 2.1.4. Aconitine Impaired *in Vivo* Tumor Growth

**Figure 4:**
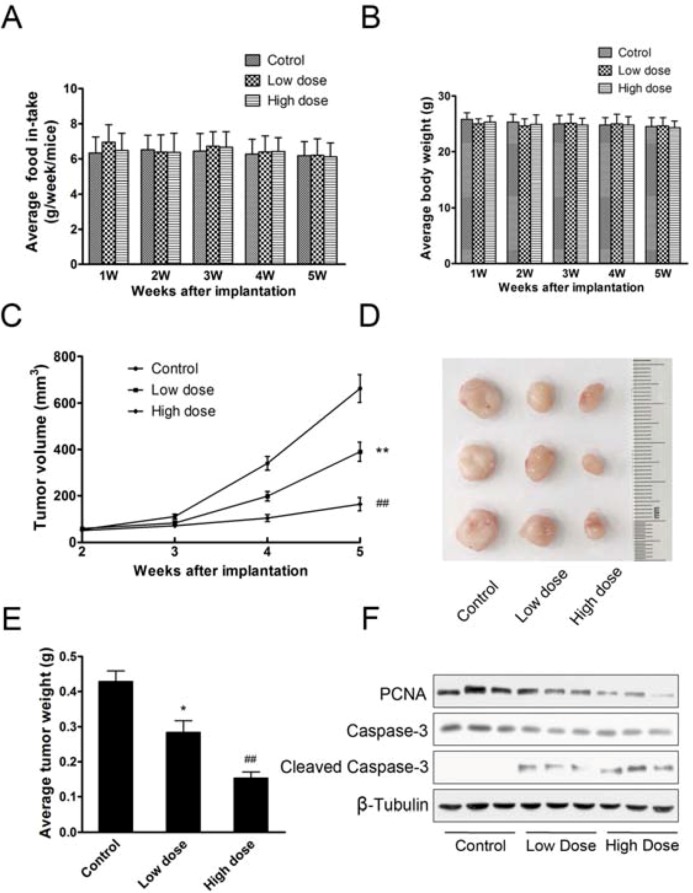
Aconitine suppressed *in vivo* tumor growth. (**A**) Average body weight of mice; (**B**) Average food in-take of mice every week; (**C**) Growth curve of tumors treated with different doses of aconitine; (**D**) Representative picture of tumors; (**E**) Average weight of tumors; (**F**) Western blotting analysis of protein expression of PCNA, Caspase-3 and Cleaved Caspase-3 in representative tumors. Data were means ± SEM. * *p* < 0.05, ** *p* < 0.01 represented that group of low dose of aconitine compared to control (0 µg/mL of aconitine), while ^##^ *p* < 0.001 represented that group of high dose of aconitine compared to control.

To further explore the role of aconitine in tumor growth, we employed an ectopic implantation model in nude mice [24]. B16 cells were subcutaneously injected into the posterior flanks of mice. When tumors reached about 5 mm in diameter, mice were divided into three groups (n = 8) and administered *per os* with different dose of aconintine of 0.12 mg/kg/d (high dose), 0.06 mg/kg/d (low dose) and saline (control), respectively. The administration of aconitine didn’t affect body weights or food intake of mice (Figure 4A,B). Compared with control, aconitine suppressed tumor growth (Figure 4C). High dose of aconitine showed much more inhibiting effect on tumor growth than low dose. Five weeks after implantation, tumors were removed and representative ones were pictured (Figure 4D). Consistent with tumor growth, tumor weights were much lighter after treatment with aconitine than control (Figure 4E). Western blotting analysis revealed that protein levels of PCNA were reduced and Cleaved Caspase-3 were enhanced (Figure 4F). It was indicated that aconitine could suppress the growth of B16 tumor cells *in vivo*. 

### 2.2. Discussion

The inhibitory effect of aconitine in some kinds of cancer, like breast cancer and lung carcinoma, has been described previously. However, there is no such report on melanoma so far [20,25]. The key finding of our study is that aconitine could inhibit the growth of B16 tumor cells both *in vitro* and *in vivo*, providing a rationale for dual targeting of PI3K/AKT and MAPK/ERK1/2 signaling to effectively control melanoma disease.

The PI3K/AKT cascade and the MAPK/ERK1/2 cascade are prototypic survival pathways that have been implicated in tumorogenesis of many cancer cells, including melanoma and thyroid cancer [15,26]. Thus, down-regulation of these pathways contributes to the inhibition of tumor development and progression, and improves responses to common therapies [16,27]. A particularly interesting finding from our studies is the role of the PI3K/AKT/mTOR signaling in the B16 cell line. We observed that inhibition of the PI3K signaling pathway at different levels with specific inhibitors had no effect on such high level of apoptosis (data not shown). Thus, aconitine possibly decreased the apoptosis of tumor cells partly by PI3K signaling pathway. The crosstalk between PI3K and ERK1/2 pathways can result in activation of one pathway if the other is inhibited singly [28,29,30]. In other words, the fact that the inhibition of PI3K or ERK1/2 alone did not induce the same levels of apoptosis as the inhibition of these two signaling pathways by treatment with aconitine showed that aconitine had more effect on anticancer than some specific inhibitors. However, we still do not know whether aconitine suppressed the pathway by direct inhibition of any of its components.

Apoptosis is a peculiar mode of cell death, regulating several physiological processes and also involved in diverse pathological conditions, defined by either deficient or excessive apoptosis. As far as cancer disease is concerned, compelling evidence indicates that a deficient apoptosis (due to alterations at different levels) may lead to tumor initiation, progression and metastases; on the other hand, the major goal of most cytotoxic anticancer agents is to commit tumor cells to apoptosis. In the present study, we use a chemosensitivity assay *in vitro* for high-throughput screening to select effective compounds to melanoma cell lines. Furthermore, we successfully established melanoma xenogrfts in nude mice and impaired tumor growth by *per os* administering aconitine *in vivo*. Aconitine, as one alkaloid that has cytotoxic activity, had shown one of the developments of anticancer therapeutics [31,32]. And aconitine inhibited the growth of three melanoma cell lines (Table 1). Thus, it will be further studied on whether the cytotoxicity of aconitine is specific to tumor cells.

On the other hand, the cancer metastasis is an important factor regarding long-term survival of patients. A key consideration in the design of antimetastatic therapeutic agents is the fact that the decreased significant numbers of disseminated tumor cells should been observed in patient’s blood, bone marrow, and distant organ sites upon initial presentation in the oncology clinic [33,34]. However, it was hard to exactly estimate the unitary effect of drugs on tumor metastasis with some drawbacks, such as animal model of tumor metastasis. In order to investigate the anti-metastatic efficacy of aconitine, orthotopic models originating from melanoma transfected cell lines expressing high-intensity fluorescent should be constructed in nude mice [35].

## 3. Experimental

### 3.1. Cell Culture and Reagents

B16, B16F1 and B16F10 mouse melanoma cells were cultured in Dulbecco’s Modified Eagle’s Medium (DMEM) containing 10% FBS, 100 units/ml penicillin, and 100 μg/mL streptomycin in a 37 °C incubator containing 5% CO_2_. Aconitine was purchased from Sigma-Aldrich (St. Louis, MO, USA), and dissolved in double distilled water as stocking solution.

### 3.2. *In Vitro* Chemosensitivity Assay

The method was performed as described previously [36]. Cells were seeded at a density of 4,000 cells per well in a 96-well plate overnight. Freshly prepared aconitine was added with the final concentration ranging from 1.6 to 100 µg/mL. Forty eight h later, cell viability was assayed by WST-1 kit (Roche, Basel, Switzerland) according to the manufacturer’s instructions. The IC_50_ value was calculated using Graphpad Prism software (Graphpad Software, Inc., San Diego, CA, USA).

B16 cells were seeded at a density of 1,000 cells per well in 96-well plates overnight, and followed by treating with 12.5 µg/mL or 6.25 µg/mL of aconitine. Cells not treated with aconitine were used as control. Cell viability was determined by WST-1 kit at indicated time points. All results were obtained from three separate experiments with six replicates per experiment.

### 3.3. Annexin-V apoptotic Assay

Apoptosis of B16 cells was measured by flow cytometry as described before [37]. Briefly, cells were trypsined, labeled with Alexa Fluor 647 Annexin V (Biolegend, San Diego, CA, USA) and 7-AAD (BD Pharmingen, Franklin Lakes, NJ, USA), and subjected to flow cytometry analysis. Cells were considered apoptotic when they were annexin V-positive and 7-AAD–negative.

### 3.4. Western Blotting Assay

Aconitine treated or non-treated B16 cells were harvested and lysed on ice for 30 min in RIPA buffer supplemented with protein kinase inhibitor cocktail. Lysates were centrifuged at 12,000 rpm for 15 min, and supernatants were collected. Total proteins were separated by SDS-PAGE, transferred onto nitrocellulose membranes in transfer buffer, and detected with antibodies against phospho-AKT (Ser-473), total AKT, phospho- ERK1/2, total ERK1/2, PCNA, Caspase-3, cleaved Caspase-3 or β-Tubulin (Cell Signaling Technology, Boston, MA, USA). Western blot was performed as described previously [38].

### 3.5. Caspase Activity

B16 cells treated or non-treated with aconitine were subjected to caspase activity assay as described before [39]. In brief, protein extracts were harvested in isolation buffer (10 mM of Tris-HCl buffer, pH 7.6, 5 mM of MgCl_2_, 1.5 mM of potassium acetate, 2 mM of dithiothreitol) supplemented with protease inhibitor cocktail (Roche Applied Science, Mannheim, Germany). General activities of caspase-3 and caspase-8 were determined by enzymatic cleavage of chromophore p-nitroanilide (pNA) from the substrates N-acetyl-Asp-Glu-Val-Asp-pNA (DEVD-pNA) and N-acetyl-Ile-Glu-pro-Asp-pNA (IEPD-pNA) (Sigma-Aldrich), respectively. The proteolytic reaction was carried out in isolation buffer containing 50 μg of cytosolic protein and 50 μM specific caspase substrate. The reaction mixtures were incubated at 37 °C for 1 h, and measured by monitoring A405 using a 96-well plate reader.

### 3.6. *In Vivo* Study

Female 6-8 weeks BALB/c nude mice were obtained from the Shanghai Laboratory Animal Center (Chinese Academy of Sciences, Shanghai, China) and maintained in special pathogen-free (SPF) condition. Animal experimental procedures were approved by the Animal Ethics Committee of the National Cancer Center. B16 cells (3 × 10^6^) were subcutaneously implanted into the posterior flanks of nude mice. Two weeks later, when xenograft tumors reached 5 mm in diameter, mice were divided into three groups (n = 8) and administered *per os* with different dose of aconintine of 0.12 mg/kg/d (high dose), 0.06 mg/kg/d (low dose) and 0 mg/kg/d (control), respectively. The duration of treatment was four weeks with five administrations per week. Mice body weights were monitored every week. The weights of food in-take were measured every day and calculated very week. Tumor volumes were monitored every week from the beginning of aconitine administration. Tumor volume was calculated as follows:

Volume = 0.5 × Length × Width^2^

Five weeks after implantation, mice were sacrificed and tumors were removed.

### 3.7. Statistical Analysis

Data in the present study were represented as means ± SEM of at least three independent experiments except specific indicated. Student’s unpaired *t* test was used for comparison between two groups. Values were considered significantly different when *p* < 0.05.

## 4. Conclusions

We have shown that aconitine could induce apoptosis in the B16 melanoma cell line *in vitro* and *in vivo*, thus decreasing the incidence of solid tumors in nude mice. Thus, aconitine merits further study as a potential anticancer drug in the future.
